# Antibiotic cement coating in orthopedic surgery: a systematic review of reported clinical techniques

**DOI:** 10.1186/s10195-021-00614-7

**Published:** 2021-12-23

**Authors:** Abdullah Ismat, Nike Walter, Susanne Baertl, Joerg Mika, Siegmund Lang, Maximilian Kerschbaum, Volker Alt, Markus Rupp

**Affiliations:** 1 Department of Trauma Surgery, University Medical Center Regensburg, Franz-Josef-Strauß-Allee 11, 93053 Regensburg, Germany; 2 Experimental Rheumatology Unit, Department of Orthopedics, Jena University Hospital, Waldkliniken Eisenberg GmbH, Klosterlausnitzer Strasse 81, 07607 Eisenberg, Germany

**Keywords:** Bone cement coating, Implant coating, Osteosynthesis, Osteomyelitis

## Abstract

**Background:**

Antibiotic-containing cement and bone graft substitute-coated orthopedic implants provide the advantages of simultaneous local antibiotic delivery and internal stable fixation, aiding in both infection eradication and osseous healing. Standardized protocols pertaining to implant coating techniques in various clinical and particularly intraoperative settings are scarce, and available literature is limited. This systematic review aims to provide a summary of the available current literature reporting on custom-made coating techniques of orthopedic implants, indications, outcomes, and associated complications in clinical use.

**Methods:**

A systematic search of the literature in PubMed, Medline, Embase, and Cochrane Library databases was performed in accordance with PRISMA guidelines. Articles reporting specifically on custom-made coating techniques of orthopedic implants in a clinical setting were eligible.

**Results:**

A total of 41 articles with a cumulative total number of 607 cases were included. Indications for treatment mostly involved intramedullary infections after previous plate osteosynthesis or nailing. A variety of implants ranging from intramedullary nails, plates, wires, and rods served as metal cores for coating. Polymethylmethacrylate (PMMA) bone cement was most commonly used, with vancomycin as the most frequently added antibiotic additive. Chest tubes and silicone tubes were most often used to mold. Common complications are cement debonding and breakage of the metallic implant.

**Conclusion:**

Adequate coating techniques can reduce the burden of treatment and be associated with favorable outcomes. Lack of general consensus and heterogeneity in the reported literature indicate that the perfect all-in-one implant coating method is yet to be found. Further efforts to improve implant coating techniques are warranted.

**Level of evidence:**

III.

## Introduction

The use of bone cement in orthopedics has become integral to many operative procedures. Its first practical use was reported around 60 years ago [1], where it was primarily used in joint replacement surgeries. Over the years, the spectrum of applications of bone cement has been growing. Developments in research and quality of bone cement as well as its delivery methods and systems have additionally contributed to its being employed as a local drug delivery agent.

Indications for cement coating include not only enhanced fixation of implants but also infection prophylaxis and treatment through local application of additive therapeutic agents including antibiotics [2]. Buchholz et al. mentioned the use of antibiotics as additives in bone cement back in 1970 [3].

Contrary to readily available antibiotic-containing products including beads, initially developed by Klemm et al. [4] in 1979, which only contain gentamicin [5], a wider range of antibiotic agents can be added to polymethylmethacrylate (PMMA) cement in accordance to the susceptibility of the causative organisms [6]. Aside from joint-replacing prosthesis, cement coating has been reported in other internal fixation methods and orthopedic implants including plates, wires, and rods [7–9].

Nevertheless, standardized clinical protocols and reports pertaining to cement coating techniques for different orthopedic implants in various operative settings are lacking in literature.

The aim of this review is to outline reported custom-made methods of cement coating techniques, indications, outcome, and complications associated with their application. The resulting insights into the particularities relevant to the various techniques should help to improve treatment delivery methods and outcomes in daily clinical practice.

## Methods

**Fig. 1:**
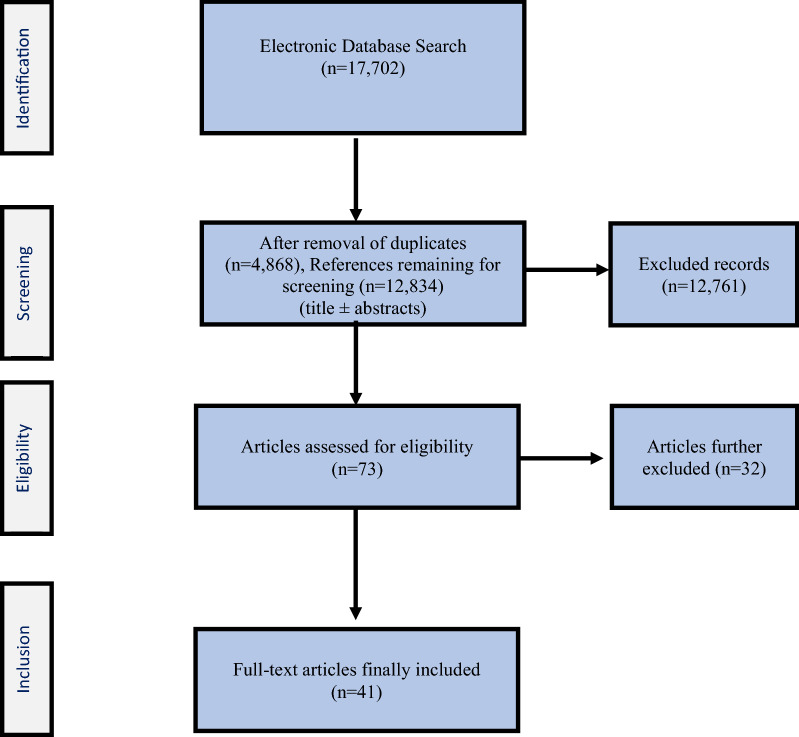
PRISMA flow diagram presenting the methodological approach for identification, screening, eligibility, and final inclusion of relevant articles

This systematic review was registered in the PROSPERO international prospective register of systematic reviews (registration no. CRD42021236015). The Preferred Reporting Items for Systematic Reviews and Meta-Analyses (PRISMA) 2009 checklist was adhered to (Fig. 1).

A search of literature reporting on coating techniques for orthopedic implants in different clinical settings was performed using PubMed, Medline, Embase, and Cochrane Library. Published articles ranging from case reports, case series, clinical trials, and review articles were initially analyzed. Articles published in English and German were reviewed. The search terms: “bone cement coating,” “bone implant coating,” “implant cement coating,” “antibiotic cement nail,” and “antibiotic cement rod” were used in the different literature databases to identify relevant articles. Articles had to include a clear outline of the custom-made cement or bone substitute coating technique applied to the orthopedic device being implanted in a clinical setting. The articles’ titles and abstracts were initially screened for relevance.

Cohort size, type of orthopedic implants coated, indications for cement coating, type of cement coating, used additives, and technical details of the cement coating techniques were noted. A table serving as a summary of each article reporting on coating techniques was then constructed to serve as a practical guide reflecting currently available evidence (Table 1). Differences in the mentioned techniques of cement coating were compared and discussed thoroughly. A total of 9118 and 8584 references were initially identifiable using the different search terms in the PubMed and Embase databases, respectively. In the Cochrane Library, the search results provided the same 2 articles under all search terms, which were not eligible for inclusion and directly excluded. Thus, a total of 17,702 (9118 from PubMed + 8584 from Embase) were identified. From this total of 17,702 references, 4868 duplicates were identified and removed, resulting in 12,834 remaining references.

**Table 1:** List of included clinical studies reporting on custom-made implant coating techniques

Authors, publication date	Article type	Cohort size	Type of implants	Indication for coating	Type of coating with or without cement	Types and amount of additives	Place of coating/cement application	Thickness of coating	Technique: digital or device assisted	Reported freedom from infection rate	Reported osseous union rate
1. Qiu et al. 2018 [9]	Case series	10 patients	Plate osteosynthesis	Postoperative infection	PMMA premixed with gentamycin	2 g vancomycin	Lateral surface of plate and bone defects	2–3 mm	Not reported	90% (9/10)	100% (10/10), 90% (9/10) without additional procedures
2. Paley et al. 2002 [10]	Case series	9 patients	3-mm beaded guidewire as a rod	Intramedullary infection	PMMA (DePuy, Inc., Warsaw, IN, USA)	2.4 g tobramycin and 2 g vancomycin per 40 g pack of bone cement	As a mold embedding the guide wire	Not available	Chest tube	100%	25% (1/4) achieved union without additional procedures and 75% of patient (3/4) with nonunions achieved union after revision
3. Fan et al. 2011 [17]	Case series	12 patients	3.2-mm Steinmann pin	Osteomyelitis after intramedullary nailing	PMMA	4 g vancomycin to each 40 g cement	Circumferential surrounding the pin	Not reported	Not reported	100% (12/12)	66.7% (8/12) without additional procedures, 100% (12/12) after revision procedures
4. Pradhan et al. 2017 [41]	Case series	21 patients	K-nail for femur	Infected nonunion of femur	PMMA (Palacos)	2 g vancomycin and 2 g gentamycin for every 40 g cement	Circumferential uniformly surrounding the nail	Not reported	manually	100% (21/21), 90% (19/21) requiring no additional surgery	85.7% (18/21) without additional procedures
5. Shyam et al. 2009 [39]	Case series	25 patients	K-nail for femur and V-nail for tibia	Infected nonunions	Not specified	2 g vancomycin and 2 g gentamicin with every 40 g cement	Circumferentially in a rolling manner	Not specified	Digitally through rolling the coating onto the implant	80% (20/25)	12% (3/25) achieved union without additional procedures, 96% (24/25) after a second procedure
6. Bharti et al. 2016 [12]	Case series	7 patients	24-gauge SS wire or 1-mm K wire and 1.8- or 2-mm Ilizarov wire	Infected nonunion of long bone	PMMA (Simplex P)	2 g cefuroxime and 2 g vancomycin	Circumferentially surrounding wires	Not reported	Using straws/Teflon tubes	85.7% (6/7)	71% (5/7) without additional procedures
7. Thonse et al. 2007 [43]	Case series	20 patients	Intramedullary nails	Infected nonunions and/or segmental bone defects	Palacos bone cement (Zimmer, Warsaw, IN)	3.6 g tobramycin and 1 g vancomycin mixed with each 40-g patch	Circumferentially surrounding the IM nail	1 mm	Metal molds	95% (19/20), with 15% (3/20) needing requiring revisions	59% (10/17) healed with no additional procedures and 100% after additional procedures
8. Thonse et al. 2008 [19]	Case series	52 patients	Intramedullary nails	Infected nonunions and/or segmental bone defects	PMMA (Palacos)	3.6 g tobramycin and 1 g vancomycin per 40 g cement	Circumferentially surrounding the IM nail	1 mm	Stainless-steel molds or silicone tubing	85% (44/52), with 73% (38/52) requiring no additional surgery	84% (41/52) total bone union rate
9. Qiang et al. 2007 [29]	Case series	19 patients	3-mm IM guidewire	IM infection after nailing	Vancomycin-impregnated bone cement (Stryker Rutherford) with 2 g vanco. with 40 g cement		Circumferentially surrounding wire		Chest tube with inner diameter similar to outer diameter of removed nails	94.7% (18/19)	21% (4/19) with no additional procedures
10. Wasko et al. 2013 [11]	Case series	10 patients	Kirschner wire	Infection after tibial IM nailing for fracture	Palacos cement	2 g gentamicin per 40-g batch	Circumferentially	Not accessible	Chest tubes 3 mm larger than the medullary canal	100% (10/10)	50% (5/10) without additional procedures, 100% (10/10) after subsequent stabilization
11. Freischmidt et al. 2020 [8]	Case series	3 patients	LCP tibia plate, femur IM nail, shoulder prosthesis	Open tibial fracture, femoral infected nonunion, septic loosening of proximal humeral nail	Cerament G, Cerament V (biodegradable biocomposites Cerament—60% fast resorbing calcium sulphate and 40% calcium hydroxyapatite)	17.5 mg gentamicin sulfate/mL paste or 66 mg vancomycin/mL paste	Implants and defects	5 mL, 10 mL, and 20 mL	Application syringe	100% (3/3)	100% (3/3)
12. Bhatia et al. 2017 [40]	Case series	20 patients	K-nail of 6 or 7 mm diameter	Infected nonunion of tibia	Not specified	2 g vancomycin and 2 g teicoplanin per 40 g bone cement	Uniformly	Coating up to 1 mm less than the diameter of last reamer	Endotracheal tube for molding	95% (19/20) with 60% (12/20) requiring no additional surgery	90% (18/20) with 60% (12/20) requiring no additional surgery
13. Selhi et al. 2012 [16]	Case series	16 patients	Not accessible	Infected nonunions of long bones	Gentamicin cement (not further specified)	4 g vancomycin mixed with 40 g gentamicin cement	Circumferentially	Not specified	Chest tubes	87.5% (14/16), 68.8% (11/16) without additional surgery	87.5% (14/16), 68.8% (11/16) without additional surgery
14. Conway et al. 2014 [20]	Case series	110 patients	Intramedullary nails	Infected nonunions	PMMA (Biomet Cobalt Bone Cement, Warsaw, Indiana)	3.6 g tobramycin and 1 g vancomycin were mixed with 40 g bone cement	Circumferentially surrounding the IM nail	From 1.25 to 5 mm	Stainless steel molds or silicone tubing	95% (105/110), 74% (81/110) without additional surgery	95% (105/110), 66% (73/110) without additional surgery
15. Reilly et al. 2016 [15]	Case series	41 patients	Threaded Ilizarov rod or ball-tipped guidewires	Infected tibial fractures after IM nail	Simplex bone cement (Stryker, Mahwah, NJ)	1–4 g antibiotics mixed with 40 g for 30 s	Circumferentially surrounding nail	Not reported	20 French chest tubes	76% (31/41)	Not applicable
16. Cho et al. 2018 [48]	Case series	40 patients	Threaded Ilizarov rod	Posttraumatic osteomyelitis and infected nonunions	Antibiotic Simplex (Stryker, USA)	4 g vancomycin and 4 g tobramycin per 40 g bone cement	Circumferentially	Not mentioned	Chest tube 1 mm less than largest reamer	85% (34/40), with 100% (40/40) after revision surgery	Not explicitly reported
17. Sancineto et al. 2008 [21]	Case series	18 patients	Ender nails, or UTN nails	Posttraumatic osteomyelitis	PMMA cement, not further specified	4 g vancomycin per 40 g cement (or gentamycin, tobramycin, or imipenem, depending on sensitivities)	Circumferentially	Not mentioned	T-95 chest tube	94% (17/18)	100% (18/18), 5.6% (1/18) without additional procedures
18. Mauffrey et al. 2014 [42]	Case report	1 patient	8.5-mm-diameter carbon-fiber nail (Carbo-Fix, Champlain, IL, U.S.A)	Femoral osteomyelitis with tibial involvement	PMMA powder containing 0.5 g gentamycin (Palacos R+G, Zimmer, Warsaw, IN, USA)	2 g vancomycin and 2.4 g tobramycin per every 40 g bone cement	Circumferentially	Not mentioned	40 Fr chest tube	Not applicable	Not applicable
19. Bar-On et al. 2010 [13]	Case series	4 patients	Kirschner wire	Chronic osteomyelitis (1 tibia and 3 femurs)	Gentamycin-impregnated PMMA cement	Not mentioned	Circumferentially	Not mentioned	28G chest tube	100% (4/4)	Not applicable
20. Bhadra et al. 2009 [30]	Case series	30 patients	Endner nails with 3.5 mm diameter	Bridging to staged IM nailing in mangled limbs, polytrauma to prevent medullary osteomyelitis and medullary osteomyelitis	Simplex cement (Stryker Orthopedics, Rutherford, NJ)	2.4 mg tobramycin, 2 g vancomycin with two packs of 40 g bone cement	Circumferentially	Not mentioned	40 Fr chest tube	Not applicable	Not applicable
21. Madanagopal et al. 2004 [31]	Case series	7 patients	Endner nails with 3.5 mm diameter	Infected tibial nails and chronic osteomyelitis of the tibia	Simplex cement (Stryker Orthopedics, Rutherford, NJ)	2.4 mg tobramycin, 2 g vancomycin with two packs of 40 g bone cement	Circumferentially	Not mentioned	40 Fr chest tube	Not applicable	Not applicable
22. Bu et al. 2020 [32]	Case series	12 patients	Elastic nail (not further specified)	Osteomyelititis after internal fixation of femoral shaft fractures	Palacos R cement	4 g vancomycin to each 40 g cement or 1.6 g gentamicin to 40 g cement in cases of *Klebsiella pneumoniae*	Circumferentially	Not reported	Silicone tube of similar diameter to IM nail	100% (12/12)	100% 12/12)
23. Makhdom et al. 2020 [22]	Case series	28 patients	10-mm-diameter IM nail (not further specified)	Infected TKA, infected tibial nonunions, ankle fusion nonunions	Simplex cement (Stryker, Kalamazoo, MI)	2 g vancomycin and 2.4 g tobramycin per 40 g cement and additional 1 g tobramycin	Circumferentially	Not mentioned	Silicone tubing with 12.7 mm internal diameter	80% (21/26)	87% (21/24)
24. Conway et al. 2015 [44]	Case series	4 patients	Internal fixation plates	3 week infected ankle ORIF, 2-month-old infected external fixation for distal radius fracture, 8-month-old infected ankle fracture ORIF, open infected olecranon fracture	Cobalt Bone Cement (Biomet Orthopedics, Inc, Warsaw, Indiana)	1 g vancomycin and 3.6 g tobramycin per 40 g cement	Circumferentially	Not mentioned	Silicone tubing	100% (4/4)	100% (4/4)
25. Anugraha et al. 2019 [26]	Case report	1 patient	Biomet hindfoot nail for tibiotalocalcaneal fusion	Osteomyelitis after hindfoot reconstruction due to Charcot neuroarthropathy	Cerament-V	10 mL Cerament-V containing 66 mg vancomycin per mL	Circumferentially	2 mm	Application syringe	Not reported	Not reported
26. Herrera-Pérez et al. 2017 [45]	Case report	1 patient	Expert-HAN (DePuy-Synthes, Spain) tibiotalocalcaneal arthrodesis nail with 10 mm diameter and 15 cm length	Secondary osteomyelitis after internal fixation of ankle fracture and subsequent spacer implantation and rupture	Hi-Fatigue G Bone Cement (Zimmer) PMMA containing 0.9 g gentamycin sulfate (0.55 g gentamycin base)	Additional 2.5 g vancomycin and 1.5 g tobramycin per 40 g bone cement	Circumferentially		12 mm sterilized silicone tube 2 mm larger in diameter than the nail	100% (1/1)	100% (1/1)
27. Liporace et al. 2012 [37]	Case report	1 patient	20-hole 4.5-mm narrow limited-contact dynamic compression plate (LC-DCP; Synthes Inc, West Chester, PA)	Infected periprosthetic femur fracture after total hip arthroplasty and subsequent ORIF	Simplex bone cement (Stryker, Mahwah, NJ)	4 vials vancomycin and 4 vials tobramycin	Entire surface of the plate	Not mentioned	Not specified	100% (1/1)	100% (1/1)
28. Mendicino et al. 2009 [33]	Case report	1 patient	0.062 Kirschner wire	Infected nonunion of tibiotalocalcaneal fusion for Charcot of hindfoot and ankle	PMMA (Stryker, Kalamazoo, MI)	1 g vancomycin per 40 g cement	Circumferentially	Not reported	Sterile surgical tubing	100% (1/1)	100% (1/1)
29. Miller et al. 2017 [38]	Case report	1 patient	Ilizarov rod with proximal locking	Infected fusion attempt after failed total ankle arthroplasty after failed internal fixation of pilon fracture	PMMA (not further specified)	3 g vancomycin and 3.6 g tobramycin per 40 g cement	Not specified	Not reported	Not specified	100% (1/1)	100% (1/1)
30. Senn et al. 2017 [28]	Case report	1 patient	Ender nail	Chronic osteomyelitis after IM nailing of tibial nonunion	PMMA (Copal G+C, Heraeus, Hanau, Germany)	2 g colistin to 40 g Copal G+C	Circumferentially	Not reported	Plastic sleeve	100% (1/1)	100% (1/1)
31. Yu et al. 2017 [34]	Case series	13 patients	4.5-mm locking compression plate	Large femoral osteomyelitis defects of more than 5 cm	PMMA (not further specified)	5 g vancomycin and 0.5 g gentamycin	On lateral surface of plate after protective filling of screw holes with sterilized bone wax	Not reported	Manually	92% (12/13) with 100% (13/13) after revision surgery	100% (13/13)
32. Liporace et al. 2014 [35]	Case report	1 patient	Two 5-mm Ilizarov rods connected via hooks and nuts	Infected periprosthetic humeral fracture after total elbow arthroplasty	Not specified	3 g vancomycin and 3.6 g tobramycin	Circumferentially	Not reported	Two chest tubes of size 36 Fr	100% (1/1)	100% (1/1)
33. Mauffrey et al. 2016 [23]	Case series	12 patients	Standard 8- or 9-mm intramedullary nail	Infected tibial nonunions with segmental bone defects ranging from 6 to 25 cm	PMMA Palacos-R (Zimmer, Warsaw, IN)	3 g vancomycin per 40 g cement. Additional 3.6 g tobramycin in presence of polymicrobial Gram-negative cultures	Circumferentially	2 mm cement mantle thickness	Chest tube with sterile mineral oil applied to its inner portion	100% (12/12)	100% (12/12)
34. Mendelsohn et al. 2013 [18]	Case report	1 patient	Threaded Steinmann pin 3 mm	Comminuted diaphyseal fracture of third metacarpal with bone loss	PMMA bone cement (not further specified)	2 g vancomycin and 2.4 g tobramycin per 40 g cement	Circumferentially	Not reported	Chest tube 28 Fr	Not reported	100% (1/1)
35. Ohtsuka et al. 2002 [46]	Case report	1 patient	Ender nail	Secondary osteomyelitis after IM nailing of an open tibial fracture	Gentamicin-containing PMMA (1.2 g gentamicin and Cemex RX/Tecres Co., Verona, Italy)	No additional antibiotics	Circumferentially	Not reported	Not specified	100% (1/1)	100% (1/1)
36. Oz et al. 2010 [14]	Case series	3 patients	K-wires	Infected nonunions of long bones	PMMA Palacos cement (not further specified)	3 g vancomycin per 40 g cement	Circumferentially	Not reported	40-Ch chest tube	100% (3/3)	100% (3/3)
37. Pruthi et al. 2020 [24]	Case series	30 patients	Intramedullary tibial nails (7–8 mm in diameter), Intramedullary femoral nails (8–9 mm in diameter), or K-nails (in 9 cases)	Infected nonunions of long bones with defects size of less than 3 cm (9 femurs, 20 tibias, 1 humerus)	Gentamicin-containing bone cement (not further specified)	4 g vancomycin per 40 g cement or 2 g vancomycin and 2.4 g tobramycin per 40 g cement	Circumferentially	2 mm in thickness	Custom-made molds. Sterile lubricant gel was used on molds before placing the cement. In cases of K-nails, manual cement application or chest tubes were used to coat	90% (27/30)	90% (27/30)
38. Gallucci et al. 2007 [36]	Case report	1 patient	Ender nail (4.5 mm)	Infected nonunion of humerus	PMMA (DePuy, Inc., Warsaw, IN, USA)	2 g vancomycin per 40 g cement	Circumferentially	Not reported	40-Fr chest tube	100% (1/1)	100% (1/1)
39. Woods et al. 2012 [25]	Case report	1 patient	Intramedullary nail with roughening of its surface using a saw or other instruments, proposed to improve cement adherence	Infected deformed ankle and hindfoot after unsuccessful tibiotalocalcaneal fusion attempt	Bone cement (not specified)	Not specified	Circumferentially	Not reported	Manually with hand-rolling on a table for uniform coating	Not specified	Not specified
40. Tomczak et al. 2019 [27]	Case series	8 patients	Hindfoot intramedullary nail (Tri-gen Hindfoot 10 mm × 160 mm, Smith and Nephew, Memphis, TN) with simultaneous use of external fixation	Clinically osteomyelitic deformed neuropathic ankles	PMMA (Simplex P with tobramycin, Stryker, Mahwah, NJ)	1 g vancomycin additionally	Circumferentially	Not reported	Sterile silicone tube	88% (7/8)	88% (7/8)
41. Dar et al. 2017 [47]	Case series	11 patients	Threaded Ilizarov rod	Infected nonunions of long bones (7 femurs, 4 tibias)	Bone cement (not further specified)	2 g vancomycin and 2 g gentamicin per 40 g or other additives as per culture results (either tobramycin, gentamicin, or amikacin; doses not further specified)	Circumferentially	Not reported	Digitally using hands to coat and create an uneven coating mantle to increase surface area	91% (10/11)	91% (10/11)

The titles and abstracts of each of the remaining references were screened for eligibility. Reviews, experimental studies, nonclinical articles, conference papers, and clinical articles reporting on precoated as well as commercially prefabricated coated orthopedic implants and ones pertaining to dental implants were excluded (*n* = 12,761). Articles reporting on use of cement or bone graft substitute to coat orthopedic implants in a clinical setting were included. This left a total of 73 articles, among which 58 full texts were available. From these 58 available full-text articles, articles reporting on and specifically outlining custom-made coating techniques as part of their surgical treatment method in an intraoperative setting were included. This resulted in the final inclusion of 41 full-text articles for appraisal (Fig. 1).

### Indications

The most common reported indications for use of cement-coated implants were intramedullary (IM) infections of long bones after previous osteosynthesis or IM nailing and infected nonunions (Table 2).

**Table 2:** Indications for antibiotic-containing cement-coated implants in different studies

Indications for antibiotic-containing cement-coated implants	Refs.
Intramedullary infections of long bones after previous osteosynthesis or nailing	[10, 11, 14, 15, 17, 21, 22, 29–32, 38, 46]
Infected nonunions	[8, 12, 14, 16, 19, 22–24, 28, 33, 36, 39–41, 44, 47, 48]
Infected total knee arthroplasty (TKA)	[22]
Segmental bone defects, infected arthrodesis, chronic osteomyelitis with bone defects after debridement, chronic infection after total knee replacements, infected Charcot ankle, infected bone after distraction osteogenesis	[19]
Early infection after internal fracture plate fixation with implant retention	[9]
Infected open fractures, loosened inverse shoulder prosthesis due to infection	[8]
Femoral osteomyelitis with tibial involvement	[42]
Chronic osteomyelitis of the tibia and femur	[13]
As part of damage control orthopedics after external fracture fixation in polytrauma patients, to prevent medullary infection during external fracture fixation	[30]
Infected fracture internal fixation, treated with coated plates	[44]
Osteomyelitis after hindfoot reconstruction for Charcot neuroarthropathy	[26]
Secondary osteomyelitis after internal fixation of ankle fracture and subsequent spacer implantation and failure	[45]
Infected periprosthetic femoral fracture after open reduction and internal fixation (ORIF) subsequent to total hip arthroplasty	[37]
Infected fusion after tibiotalocalcaneal fusion attempt	[26, 38]
Infected deformed ankle and hindfoot after unsuccessful tibiotalocalcaneal fusion attempt	[25]
Deformed neuropathic ankles with clinical osteomyelitis	[27]
Large femoral osteomyelitis defects with a size exceeding 5 cm after debridement	[34]
Infected periprosthetic humeral fracture after total elbow arthroplasty	[35]
Tibial nonunions with segmental bone defects ranging from 6 to 25 cm, with an average size of 13 cm	[23]
Comminuted diaphyseal fracture of third metacarpal with bone loss	[18]

Antibiotic-impregnated cement-coated implants were also used to treat chronic osteomyelitis with and without bone defects after debridement. Chronic infections after total knee replacements and shoulder prosthesis have also been treated with coated implants. Additionally, this treatment method was used to treat chronic osteomyelitis and infected Charcot ankles.

In the acute setting, indications for cement-coated implants included infected open fractures, early infections following fracture plate osteosynthesis, and treatment of polytrauma patients requiring external fixation as part of damage control orthopedics and for medullary infection prevention.

### Coating techniques

Various orthopedic implants have been used as metal cores in the coating process (Table 3). Early reports, published almost 20 years ago, utilized intramedullary guidewires of 3 mm thickness [10]. This allowed for some stability across fracture sites and infected bones but was limited and inadequate for weight-bearing and definitive bone healing. Other metal cores, also of limited stability, used included K-wires [11–14], Ilizarov wires [12, 15], steel wires [16], ball-tipped guide wires [15], and Steinmann pins [17, 18]. To provide more stable constructs and allow for simultaneous weight-bearing, other groups reported on the use of IM nails [19–25] of long bones for coating. This mainly included antegrade and retrograde femoral IM nails as well as tibial IM nails. Furthermore, clinically beneficial constructs were also created with use of knee and ankle arthrodesis implants [19, 26], and some authors even reported coating of inverse shoulder prosthesis and plates used for revisions of septic loosening and for fracture fixation [8], respectively. To provide more protection across the fusion site and aid in early weight-bearing, some authors reported using antibiotic-containing coated IM nails in combination with simultaneous external ring fixation in cases of unstable infected neuropathic ankles in obese patients [27].

**Table 3:** Various implants used

Implant used as metal core	Refs.
3-mm beaded intramedullary guidewire	[10, 29]
Femoral antegrade and retrograde nails (not further specified)	[8, 19]
Tibial nails (TriGen intramedullary nails, Smith and Nephew, Memphis, Tennessee), knee arthrodesis (not further specified), ankle arthrodesis (not further specified)	[19]
Tibial nail (UTN, Synthes, Oberdorf, Switzerland)	[21]
10-mm IM nail (not further specified)	[22]
Intramedullary nail (not further specified)	[25]
Intramedullary tibial nails (7–8 mm in diameter), intramedullary femoral nails (8–9 mm in diameter), or K-nails (in 9 cases)	[24]
Küntscher nails	[16, 39–41]
V-nails for tibia (not further specified)	[39]
Steinmann pin	[17]
K-wires	[11–14]
0.062 K-wire or Steinmann pin	[33]
Threaded Steinmann pin of 3 mm diameter	[18]
3.5-mm Ender nails	[21, 30, 31]
4.5-mm Ender nail	[36]
Ender nail (not further specified)	[28, 46]
1.8- or 2-mm Ilizarov wires	[12, 15]
6-mm Ilizarov rod	[48]
Ilizarov rod (not further specified)	[38, 47]
Two 5-mm Ilizarov rods connected via hooks and nuts	[35]
Plate osteosynthesis (not further specified)	[9]
Low compression plate (LCP) for tibia, Intramedullary femoral nail (LFN 360/16 mm, Fa Synthes), inverse shoulder prosthesis (Fa Synthes DePuy)	[8]
Steel wires	[16]
Ball-tipped guide wires	[15]
Radiolucent 8.5-mm-diameter carbon-fiber nail (Carbo-Fix, Champlain, IL, USA)	[42]
Radiolucent carbon-fiber intramedullary nail, 10 mm (Carbofix, Orthopedics, Herzliya, Israel)	[23]
Elastic nail (not further specified)	[32]
Internal fixation plates for fibula, radius, and olecranon	[44]
Biomet hindfoot nail for tibiotalocalcaneal fusion	[26]
Expert-HAN (DePuy-Synthes, Spain) tibiotalocalcaneal arthrodesis nail with 10 mm diameter and 15 cm length	[45]
20-hole 4.5-mm narrow limited-contact dynamic compression plate (LC-DCP; Synthes Inc, West Chester, PA)	[37]
4.5-mm locking compression plate	[34]
Hindfoot intramedullary nail (Tri-gen Hindfoot 10 mm × 160 mm, Smith and Nephew, Memphis, TN)	[27]

### Type of bone cement/bone graft substitute and quantity of antibiotic additives used in different studies

PMMA cement was the bone cement most commonly used (*n* = 34) to coat orthopedic implants in a custom-made fashion, with Palacos (*n* = 7) and Simplex (*n* = 7) formulations being the most frequently used and mixed with antibiotic additives (Table 4). Some studies (*n* = 6) also utilized premixed bone cement formulations containing most commonly premixed gentamicin (*n* = 6) or premixed tobramycin (*n* = 1). More recently, certain groups [8, 26] reported on the application of Cerament (Bonesupport AB, Lund, Sweden) as a coating, a bone graft substitute consisting of calcium sulfate and calcium hydroxyapatite, premixed with either gentamicin or vancomycin.

**Table 4:** Summary of type of cement or bone graft substitute and type and quantity of antibiotic additives used for the coating mantle

Bone cement/bone substitute (± premixed antibiotics)	Antibiotic additives	Refs.
PMMA (DePuy, Inc., Warsaw, IN, USA)	2.4 g tobramycin and 2 g vancomycin per 40 g, vacuum mixing	[10]
PMMA (Stryker Rutherford)	2 g vancomycin per 40 g	[29]
PMMA, Palacos (Zimmer, Warsaw, Indiana)	3.6 g tobramycin and 1 g vancomycin mixed with each 40-g patch of Palacos	[19]
Not specified	2 g vancomycin and 2 g gentamicin mixed per 40 g bone cement	[39]
PMMA cement (Simplex; Howmedica, Rutherford, NJ)	4 g vancomycin added to each 40 g PMMA cement	[17]
PMMA, Palacos (Heraeus Kulzer, Hanau, Germany)	2 g gentamycin per batch of Palacos cement (total two batches used)	[11]
PMMA, Simplex P bone cement	2 g cefuroxime and 2 g vancomycin added per 40 g Simplex P bone cement	[12]
PMMA, not further specified	2 g vancomycin and 2 g teicoplanin with each 40 g bone cement	[40]
PMMA, Palacos	2 g vancomycin and 2 g gentamycin per 40 g cement	[41]
PMMA (Smith and Nephew, TN, USA) (premixed with gentamicin)	2 g vancomycin with one batch of premixed gentamicin-containing PMMA cement	[9]
Bone cement (not further specified) (premixed with gentamicin)	4 g vancomycin mixed with 40 g gentamicin cement	[16]
PMMA (Biomet Cobalt Bone Cement, Warsaw, IN)	3.6 g tobramycin and 1 g vancomycin mixed with 40 g bone cement	[20]
Simplex bone cement (Stryker, Mahwah, NJ)	1–4 g antibiotics mixed with 40 g Simplex for 30 s before injecting	[15]
PMMA, Simplex (Antibiotic Simplex, Stryker USA)	4 g vancomycin and 4 g tobramycin with 40 g bone cement	[48]
PMMA (Palacos R+G, Zimmer, Warsaw, IN, USA) (premixed with 0.5 g gentamicin)	2 g vancomycin and 2.4 g tobramycin were each mixed with 40 g PMMA containing 0.5 g gentamicin	[42]
PMMA cement (not further specified) (premixed with gentamicin)	No additional additives further specified	[13]
PMMA bone cement (Simplex, Stryker Orthopedics, Rutherford, NJ)	2.4 g tobramycin and 2 g vancomycin with two packs of 40 g PMMA	[30]
PMMA bone cement (Simplex, Stryker Orthopedics, Rutherford, NJ)	2.4 g tobramycin and 2 g vancomycin with two packs of 40 g PMMA	[31]
PMMA, not further specified	4 g vancomycin (or gentamycin, tobramycin, or imipenem, depending on culture results) per 40 g PMMA cement	[21]
PMMA bone cement (PALACOSR)	4 g vancomycin or 1.6 g gentamicin per 40 g PMMA	[32]
PMMA bone cement (Simplex, Stryker, Kalamazoo, MI)	2 g vancomycin and 3.6 g tobramycin per 40 g PMMA	[22]
PMMA bone cement (Cobalt, Biomet Orthopedics, Inc., Warsaw, IN)	1 g vancomycin and 3.6 g tobramycin per 40 g PMMA	[44]
Hi-Fatigue G Bone Cement (Zimmer) PMMA [premixed with 0.9 g gentamycin sulfate (0.55 g gentamycin base)]	Additional 2.5 g vancomycin and 1.5 g tobramycin per 40 g bone cement	[45]
Simplex bone cement (Stryker, Mahwah, NJ)	4 vials vancomycin and 4 vials tobramycin	[37]
PMMA (Stryker, Kalamazoo, MI)	1 g vancomycin per 40 g cement	[33]
PMMA (not further specified)	3 g vancomycin and 3.6 g tobramycin per 40 g cement	[38]
PMMA (Copal G+C, Heraeus, Hanau, Germany) (premixed with 1 g gentamicin and 1 g clindamycin per 40 g)	2 g colistin per 40 g cement	[28]
PMMA (not further specified)	0.5 g gentamycin and 5 g vancomycin per 40 g cement	[34]
Bone cement not specified	3 g vancomycin and 3.6 g tobramycin per 40 g cement	[35]
PMMA Palacos-R (Zimmer, Warsaw, IN)	3 g vancomycin per 40 g cement Additional 3.6 g tobramycin in presence of polymicrobial Gram-negative cultures	[23]
PMMA (not further specified)	2 g vancomycin and 2.4 g tobramycin per 40 g cement	[18]
PMMA (Cemex RX/Tecres Co., Verona, Italy) (premixed with 1.2 g gentamicin)	No additional antibiotics	[46]
PMMA Palacos cement (not further specified)	3 g vancomycin per 40 g cement	[14]
Bone cement (premixed with gentamicin) (not further specified)	4 g vancomycin per 40 g cement or 2 g vancomycin and 2.4 g tobramycin per 40 g cement	[24]
PMMA (DePuy, Inc., Warsaw, IN, USA)	2 g vancomycin per 40 g cement	[36]
Bone cement (not further specified)	Not specified	[25]
PMMA (Simplex P, premixed with tobramycin, Stryker, Mahwah, NJ)	1 g vancomycin mixed additionally	[27]
Bone cement (not further specified)	2 g vancomycin and 2 g gentamicin per 40 g cement or other additives as per culture results (either tobramycin, gentamicin, or amikacin; doses not further specified)	[47]
Cerament G, Cerament V (premixed with either vancomycin or gentamicin)	5 mL Cerament G (17.5 mg gentamicin sulfate/mL paste), 10 mL Cerament V (66 mg vancomycin/mL paste)	[8]
Cerament V (premixed with vancomycin)	10 mL Cerament V (66 mg vancomycin/mL paste)	[26]

Antibiotic additives which were mixed with the bone cement to form the coating mantle of orthopedic implants most commonly involved the use of vancomycin, either alone (*n* = 7) or in combination with other antibiotic agents (*n* = 24). Vancomycin was most frequently mixed with tobramycin (*n* = 16) followed by gentamicin (*n* = 6), teicoplanin (*n* = 1), and cefuroxime (*n* = 1). Additionally, one report mentioned the use of PMMA premixed with gentamicin and clindamycin (Copal G+C, Heraeus, Hanau, Germany) with an extra 2 g colistin added to provide local antibiotic treatment to medullary infection with multiresistant *Pseudomonas aeruginosa* [28].

### Reported molding techniques and instruments

Instruments used as molds to coat the various implants serving as the metal core of the antibiotic-impregnated cement-coated constructs mainly consisted of chest tubes and silicone tubes of different sizes in accordance to the authors and their desired construct to be implanted (Table 5). Manual or digital application of the coating was used for different implants ranging from rods, plates, nails, and pins. Some authors even used food straws and Teflon tubes (*n* = 1), while others utilized endotracheal tubes (*n* = 1) or metal molds (*n* = 3).

**Table 5:** Reported molding techniques outlined in detail

Application and molding techniques of coating (digital, manual, device assisted)	Refs.
Chest tubes	[10, 11, 13–16, 18, 21, 23, 24, 29–31, 35, 36, 42, 48]
Silicone tubes	[12, 19, 20, 22, 27, 28, 32, 33, 43–45]
Manually with digital hand-rolling	[25, 39, 41]
Manual application without mention of hand-rolling	[9, 17, 34, 47]
Manual application using a syringe	[8, 26]
Steel/metal molds	[20, 24, 43]
Food straws, Teflon tubes	[12]
Endotracheal tubes	[40]
Not specified	[37, 38, 46]

### Treatment strategies and outcomes

Treatment with antibiotic-containing cement- or bone graft substitute-coated orthopedic implants was not only performed as the initial revision procedure to aid in eradication of infection with simultaneous limited construct stability later needing further revision surgery for definitive fixation [10–14, 21, 23, 29–38] (*n* = 17), but also as the main definitive revision procedure with either no further planned procedures [8, 9, 15, 17, 19, 20, 22, 24–26, 28, 39–45] (*n* = 18) or additional surgery solely to remove the coated implants after completed healing and controlled infection [16, 46, 47] (*n* = 3). Some studies (*n* = 2) also implemented antibiotic-containing cement-coated implants as part of a three-stage revision procedure protocol [18, 48]. Others (*n* = 1) used intramedullary coated nails with simultaneous external ring fixation, the latter of which was to be removed after radiological bone healing was seen [27].

In cases where antibiotic-containing cement-coated implants were indicated and used as the definitive single-stage surgical procedure, 10–88% of them needed additional revision procedures either to control infection or to achieve bony union. The majority (16/18) had a required revision rate of 50% or less.

### Complications and their management

Specific complication rates resulting from antibiotic-containing cement-coated implants ranged from 5% to 30% across different studies. This mainly involved nail-cement debonding, nail breakage, nail bending, and migration, occurring in 10–30% of cases in a series of 20 patients [40]. Further reported complications included joint stiffness, septic arthritis, and more rarely, local antibiotic intolerance due to hypersensitivity [21]. The authors of that report, did not further specify how the hypersensitivity was clinically evident. Nail-cement debonding was more commonly encountered during nail removal and occasionally during nail insertion [20]. Management of this specific complication ranged from use of certain instruments and extraction tools such as J-hooks and additional reaming [43] to creation of a cortical window to aid in cement retrieval [16]. Infection of neighboring joints, suspected to be related to nail insertion site contamination, was managed by using an extracapsular nail insertion point [21]. Nevertheless, the majority of authors did not report any specific complications when using antibiotic-containing cement-coated implants (Table 6).

**Table 6:** Encountered complications

Encountered complications reported	Refs.
Broken antibiotic cement nail/rod	[10, 14, 20, 29, 40, 41]
Cement–nail debonding	[19, 20, 43]
Cement cracking	[10]
Nail migration	[40]
Distal locking screw migration	[22]
Nail bending	[40]
Difficult nail removal	[16, 19, 20, 29, 40, 41]
Adjacent knee-joint infection	[29]
Septic hip arthritis likely from insertion site contamination	[21]
Limited range of knee-joint motion	[39]
Knee stiffness	[41]
Union failure	[16, 22, 24, 47]
Persistent and/or recurring infection	[22, 24, 34]
Amputation	[22, 27, 29, 43]
Nerve compression, painful screw, hematoma, skin infection, and joint contractures	[20]
Painful olecranon plate needing removal	[44]
Local antibiotic intolerance related to vancomycin hypersensitivity	[21]
Pin-site infection, wound dehiscence, proximal tibial fracture	[27]
No specific complications reported	[8, 9, 11–13, 15, 17, 18, 23, 25, 26, 28, 30–33, 35–38, 42, 45, 46, 48]

## Discussion

After a thorough appraisal of the available literature reporting specifically on antibiotic-containing cement and bone graft substitute coating techniques for orthopedic implants in an operative clinical setting, 41 articles with a collective total number of 607 cases were identified. Available reviews related to this topic are scarce and have so far focused on general indications, efficacy, and outcomes with no specific detailed description of the different coating techniques and their associated particularities in an intraoperative setting [49–51].

To date, there are no general consensus and specific guidelines on the particular techniques used to cement-coat implants in a custom-made fashion. The available evidence is limited to case reports and case series from different groups reporting on their custom-made coating techniques as part of different treatment protocols in various clinical settings. Some authors used this treatment method not only as part of a staged treatment protocol with the initial aim of infection control followed by definitive fixation for bone healing but also as the sole surgical procedure to treat infected nonunions and posttraumatic osteomyelitis [17, 19, 39–41].

Management of infected nonunions and posttraumatic osteomyelitis is multifactorial and involves several components mainly consisting of removal of infected hardware, adequate thorough debridement of infected bone and tissues, appropriate dead space management with local antibiotic delivery to control infection, and, if necessary, adequate soft tissue coverage and bone defect reconstruction [10, 52]. Use of local antibiotic delivery methods, with reported results comparable to treatment with systemic antibiotics alone [53, 54], can help reduce the burden of toxicity associated with systemic antibiotics and address poor penetration from poor vasculature and biofilm formation at the site of infections as well as potential development of antibiotic resistance [40].

Aside from high concentrations of antibiotics needed to control infection, bone stability must also be provided for adequate bone healing and union to occur, particularly in cases of infected fractures or nonunions. This generally entails a staged treatment protocol with usually two planned procedures being necessary with provisional stability being provided through means of external fixation, casts, or splints [12, 40]. However, complications such as pin-site infections, joint stiffness, contractures, and others related to patient compliance limit the usefulness of external fixation [55].

To reduce the burden of treatment and improve outcomes, antibiotic-impregnated cement-coated nails, offering both local antibiotic delivery and adequate internal bone stability allowing for simultaneous control of infection and osseous union, were introduced around 20 years ago [10]. However, and as evident in this review, there is no general uniform method of applying this form of surgical treatment. Accordingly, an individualized treatment strategy can be tailored as necessary.

To provide the required stability for osseous union across the site of infected bone, various implants were used as the metal core of the antibiotic-containing cement-coated construct. Because guidewires, K-wires, Ilizarov wires, and nails such as Ender or Küntscher nails do not necessarily offer the stability required for bone healing, intramedullary nails have been coated and implanted as means of definitive internal fixation [19]. This not only avoids the need for external stabilizing systems but can also, more importantly, allow for weight-bearing, which in turn improves outcomes and reduces complications [19]. From an antibiotic elution properties perspective, coated interlocking nails have been shown to have better antibiotic delivery characteristics in comparison with coated guidewires, potentially from a thinner cement mantle and cooler associated exothermic reactions [56], further supporting their use particularly when a definitive one-stage procedure with the needed adequate mechanical stability is indicated. Choice of the particular implant to be coated should be made on an individual, case-dependent basis considering both patient characteristics and anatomical particularities. In addition to intramedullary nails, arthrodesis nails, plates, and joint replacement prosthesis have also been coated with antibiotic-containing cement, with favorable outcomes. More recently, carbon-fiber nails [42] have been applied as they are radiolucent and reduce the production of artifacts, particularly on radiological follow-up with magnetic resonance imaging (MRI), thus allowing for more accurate treatment monitoring. However, radiological follow-up by MRI is not standard, and its clinical use after surgical interventions remains questionable. Interestingly, with regards to bacterial adhesion on implant surfaces, carbon-fiber rods did not demonstrate inferior results compared with steel rods in an experimental study potentially supporting their use as a sound alternative option to conventional metallic implants [57].

The most specific reported complication potentially arising after use of cement-coated implants is nail–cement debonding. In the largest series, involving more than 100 patients, Conway et al. [20] reported encountering this complication in 23 from a total of 110 cases (~21%) during both insertion and removal of the antibiotic-containing cement-coated implants. Other authors encountered this complication in approximately 10–30% of cases [10, 19, 29, 40, 41, 43]. Management mainly involved removal of retained debonded cement with use of certain extraction tools, such as J-hooks from hip arthroplasty instrumentation set [43], and additional reaming. In some cases, creation of a cortical window to facilitate retrieval may be necessary [16]. Moreover, use of a threaded metal core has been suggested to prevent occurrence of cement debonding upon removal [49], and in one report the authors proposed roughening the surface of the IM nail using saws and other instruments before coating to improve cement adherence [25].

Different antibiotic agents have been used as additives to mix with bone cement in accordance with susceptibility testing [6]. Vancomycin was the most commonly used antibiotic in all studies; this corresponds with methicillin-resistant *Staphylococcus aureus* (MRSA) being the most frequent causative pathogen [20]. Aminoglycosides, such as gentamicin, have also been shown to be heat-stable with low allergic potential, making them suitable as antibiotic additives [58]. Different bone cement formulations have been associated with various elution properties when certain antibiotics are added to them, with Palacos bone cement having generally more favorable properties in comparison with Simplex bone cement [59–61]. Furthermore, use of a combination of different antibiotic agents, additionally mixed, was shown to improve elution properties [62, 63]. PMMA bone cement was most commonly used to prepare the coated implant. Moreover, custom-made intraoperative addition of antibiotics to the cement formulation has been shown to provide better elution properties in comparison with commercially premixed antibiotic-containing cement preparations [64, 65], further supporting custom-made intraoperative preparation of these constructs in accordance with antimicrobial susceptibility testing. More recently, bone graft substitutes, composed of calcium phosphate and calcium hydroxyapatite, premixed with either vancomycin or gentamicin have been utilized to coat orthopedic implants [8, 26]. Comprehensive evidence elaborating on the microbiological and biomechanical properties of such bone graft substitutes in the coating of orthopedic implants, to treat infected fractures and nonunions, in comparison with the conventional method using bone cement is still very limited, and lately encouraging results have been reported when CERAMENT G was applied as part of a one-stage treatment protocol of chronic osteomyelitis [66].

Molding techniques varied across different studies. A uniform circumferentially applied antibiotic-containing cement mantle was created either manually, through rolling, or with the aid of certain molds. Thonse et al. [19] introduced the silicone tubing technique and reported better and more time-efficient coating with the use of this method as opposed to the previously used stainless-steel molds. Most other groups used chest tubes as a molding instrument. Retrieval of the coated nail was usually performed after the cement was allowed to set [19]. Some authors submerged the construct in a bowl containing cool sterile water to prevent heat accumulation and potential plastic tube melting during the exothermic phase [67]. To avoid breakage of the cement mantle during retrieval of the coated nail before insertion, sterile mineral oil was used to lubricate the inner mold surface, allowing for faster fabrication time and easier tube removal [68]. With regards to the antibiotic elution properties, one study showed no difference in the elution properties of tobramycin with or without use of mineral oil [69].

In cases where bone defects are present after thorough debridement, treatment with antibiotic-containing cement-coated nails has been associated with varying results. To assess the efficacy of cement-coated implants within the scope of revision surgery to surgically treat infected nonunions with bone defects and remaining in situ fixators, Shyam et al. [39] conducted a study involving 25 patients. The reported outcomes from this study demonstrated more unfavorable results with increasing size of the bone defect left after debridement of infected nonunion to be treated and suggested the use of alternative treatment methods when defects exceeded 6 cm in size. In this report, patients with large defects required additional surgery in the form of debridement and application of an Ilizarov ring fixator. Four out of five patients achieved union. One patient developed a stiff nonunion and was mobilized with a brace after declining further surgery [39]. On the contrary, Mauffrey et al. [23] reported good outcomes with use of antibiotic-containing cement-coated intramedullary nails in the two-stage treatment of 12 patients with infected tibial nonunions and segmental bone defects ranging from 6 to 25 cm in size. Moreover, Yu et al. [34] demonstrated a 100% union rate and a 92% freedom from infection rate in a sample of 13 patients with chronic femoral osteomyelitis and remaining defects exceeding 5 cm in size after debridement, and a mean defect size of more than 9 cm, when using antibiotic-containing cement-coated plates for the first stage as part of a two-stage induced membrane treatment protocol. Thus, more elaborate evidence is needed to conclude as to which antibiotic-containing cement-coated construct, consisting of either intramedullary nails or plates, is more effective in the two-stage treatment of large bone defects.

Overall, high rates of infection control and bony union are possible with the use of antibiotic-containing cement-coated implants in particular clinical settings. Outcomes and major complications, especially when used in form of a one-stage definitive procedure, have been comparable between the reporting studies with variable patient collectives. Higher rates of infection control were achievable through use of antibiotic-containing cement-coated implants than osseous union rates. Bony union rates increased and ranged from 70% to 100% of cases after additional procedures involving exchange nailing and bone grafting were performed. Despite the mentioned complications, the results presented in this review can be regarded as advantageous when using this form of surgical treatment when indicated.

## Conclusions

Cement coating of orthopedic implants is supportive and sometimes necessary in various clinical settings. Adequate cement coating techniques can reduce the burden of treatment and be associated with favorable outcomes, particularly in revision surgery. Downsides observed with current cement coating techniques are debonding of cement during implant removal and breakage of the coated metallic implant. After reviewing the available evidence reporting on different custom-made cement coating techniques with their respective benefits and limitations applied so far, it is evident that the perfect all-in-one implant cement coating method has yet to be found, and that a reasonable amount of heterogeneity is present in reported literature. Further prospective targeted research on these cement coating methods in different operative settings is warranted to better optimize patient care and outcomes when applying these techniques.

## Data Availability

The datasets used and/or analyzed during the current study are available from the corresponding author on reasonable request.
